# Meaningful crime prevention or just an ‘Act’:Discourse Analysis of the criminalisation of contract cheating services in Australia

**DOI:** 10.1007/s10611-022-10025-2

**Published:** 2022-04-01

**Authors:** Andrew Groves, Victoria Nagy

**Affiliations:** 1 Flinders University, College of Business, Government and Law, Adelaide, Australia; 2 Criminology, College of Business, Government and Law, Ring Road, Bedford Park, 5042 Australia; 3 University of Tasmania, School of Social Sciences, Hobart, Australia

**Keywords:** Contract cheating, Criminalisation, Australia, Politics, COVID-19, Political discourse analysis

## Abstract

Contract cheating remains a significant problem for universities and higher education (HE) generally, both within Australia and internationally. In 2020, the Australian Federal Government passed legislation establishing a new criminal offence, criminalising the provision or advertisement of academic cheating services by individuals and businesses. This legislation represents the Australian Government’s formal commitment to a criminal justice response to address the problem of contract cheating behaviour, which seeks to prevent and minimise the use and/or promotion of such cheating services within the higher education sector. This paper provides a political discourse analysis (PDA) and interpretive policy analysis (IPA) of Australian Parliamentary Hansard documents regarding debate of the *Tertiary Education Quality and Standards Agency Amendment (Prohibiting Academic Cheating Services) Bill 2019.* Our findings suggest a discord between the putative purpose of this legislation and the way the contract cheating problem has been represented in Australian Parliament. We argue that debates regarding the solution to, or at least how to address contract cheating first need to understand and agree on the problem if they are to meaningfully prevent crime. Our analysis exposes the politicisation of the higher education sector and associated discourse, where concern about contract cheating, in this case, was used as a vehicle to further rationalise ongoing Government paternalism and interference in tertiary institutions, underscoring the need for critical evaluation of criminological interventions.

## Introduction

The ‘problem’ of university students’ engagement in contract cheating or assignment outsourcing has been widely researched in Australia (see Clare et al., 2017; Baird & Clare, 2017; Bretag et al., 2019; Nagy & Groves, 2021; Awdry, 2020) and internationally (Bowers, 1964; McCabe & Trevino, 1997; McCabe, 2005; Hughes & McCabe, 2006a, 2006b; Clarke & Lancaster, 2006; Walker & Townley, 2012; Lancaster, 2020). This research has been multi-disciplinary, though comparatively little from criminology (Nagy & Groves, 2021), largely attributable to the assumption that academic misconduct is ‘innocuous’ or harmless (Smith et al., 2013:89). Contract cheating represents a serious form of academic misconduct, involving students either outsourcing the completion of assignments to another person or third-party for submission as their own work, or having others sit examinations or practical tests on their behalf, often for payment within commercial arrangements (Clarke & Lancaster, 2006; Clare et al., 2017; Curtis & Clare, 2017; Awdry, 2020). As noted by Walker and Townley (2012:27), engagement of third-parties, particularly within formal commercial arrangements, is concerning for educators because it is extremely difficult to detect, constitutes fraud, and seemingly exposes a series of broader problems within HE (see Sommerville, 2021). Contract cheating emphasises universities’ vulnerability to threats against academic integrity, the quality and value of qualifications and the reputation of Australia’s tertiary education sector, both domestically and internationally. Furthermore, students have been found to outsource their assignments in diverse ways, often not involving monetary payments, with use of “essay mills, bespoke assignment services, essay bidding services, peer-to-peer file sharing sites … and obtaining work from other students, colleagues, friends and family members” (Awdry, 2020:1). While contract cheating is not new, occurring from the 1970s in various settings with attempts made in the US to curb contract cheating through legal means (see Clare et al., 2017; McCormick & Whaley, 2014), it is only in the last decade that it has garnered considerable scholarly and media attention (Awdry, 2020; Curtis & Clare, 2017; Lancaster, 2020).

Several countries have indicated concern about contract cheating as an “…emerging threat to higher education”, including the US, Canada, New Zealand, the UK and others in Europe (TEQSA, 2017:iii; QAA, 2017). A relatively recent but paradigmatic shift in the conceptualisation of and response to contract cheating has been its formalisation within criminal justice policy. In the last decade, an increasing number of jurisdictions have employed legal approaches to prohibit/outlaw contract cheating, intended to identify, punish, and deter such misconduct (Amigud & Dawson, 2020; Draper & Newton, 2017). In September 2020, the Australian Federal Government joined this movement, passing legislation making it an offence to provide or advertise academic cheating services in HE (Cosenza, 2020). Amending the *Tertiary Education Quality and Standards Agency Act 2011* (herein ‘TEQSA Act 2011’), the *Tertiary Education Quality and Standards Agency Amendment (Prohibiting Academic Cheating Services) Act 2020* (Cth) (herein ‘TEQSAA(PACS) Act 2020’) explains key terms, the expanded role of the regulatory body (TEQSA) and the nature and scope of both criminal and civil penalties linked to the provision and advertisement of contract cheating services, for either commercial or non-commercial purposes.

Notwithstanding support for the criminalisation of contract cheating – which naturally draws the attention of criminology – concerns about its effectiveness abound. Despite these concerns, this article does not seek to predict or evaluate the effectiveness of this legislation but to illuminate the discord in the political debate leading to the passing of the Act and challenge the appropriateness of this legislation. Examination of the debate surrounding this contract cheating legislation fails to address these broader concerns because it is not problem-oriented, instead focusing on opportunity reduction. Based on our analysis, the criminalisation of contract cheating mirrors the political discourses and responses to other criminal justice ‘problems’ where arguably complex issues are sought to be addressed with a one-size-fits-all approach. While reducing the opportunity for students to engage contract cheating services is one facet of the response to the problem, it risks displacing contract cheating into other avenues that this legislation does not cover as it is not context sensitive (Cherney, 2006). The law is principally reactive, criminalising those who provide or advertise cheating services once cheating has occurred or attempting to block access to specific sites that are overt in their contract cheating services, rather than examining the demand for and socio-cultural determinants regarding use of these services, which – as many in the academy argue (Awdry, 2020; Lancaster, 2020; Newton, 2018) – should be the priority. Analysis of the parliamentary debates about the initial Bill establishes that Australian parliamentarians cannot articulate what problem they are attempting to solve. We argue in this paper that there are many ‘problems’ identified in the debate regarding contract cheating, but no one, comprehensive issue that is likely to be solved by this legislation. This raises the question of what the criminalisation of contract cheating seeks to achieve, specifically whether it represents a meaningful effort to employ the longstanding scholarship of crime prevention, or whether it is just an ‘Act’, part of the broader politicisation of Australian HE?

## Background and literature review

### Prevalence and Perspectives

The prevalence of contract cheating is extremely difficult to determine. While essay mills, peer-sharing sites, and bespoke assignment providers are increasingly accessible to students, through both simple Internet searches and exposure to targeted advertising on frequently used websites including social media, research suggests increased accessibility may not beget greater prevalence (Clare et al. 2017; Rundle et al., 2019). Some scholars claim, “the prevalence of contract cheating is unknown” (Walker & Townley, 2012:27), while many consider this too definitive, arguing “that *little* is known” (Curtis & Clare, 2017:116, emphasis in original), and further still, others call for expanded scholarly investigation (see Wallace & Newton, 2014). Estimates of prevalence have varied widely in empirical studies, contributing to a complex domestic and international milieu. Despite seemingly increased accessibility to cheating services, Australian data suggests a 10-year decline (from 3.5 percent) to 2.8 percent in 2014 (Curtis & Vardanega, 2016). International estimates have similarly varied, ranging from one percent (Maxwell et al., 2006) to 7.9 percent (Zafarghandi et al., 2012), with recent work suggesting that more than 15% of students worldwide (approx. 31 million) have outsourced assignments (see Newton, 2018; Awdry, 2020). Despite these empirical variations, it is accepted that contract cheating represents a significant and growing concern within HE (Clare et al., 2017; Lancaster, 2020; Newton, 2018). Indeed, several recent largescale examples in Australia, notably the MyMaster scandal but also others (McNeilage & Visentin, 2014; Visentin, 2015; see also Smith, 2015), have underpinned the ‘ratcheting up’ of institutional responses, including within one of the authors’ universities (see Jacks, 2016; Delibasic & Royall, 2021).

Criminological studies have applied diverse theoretical perspectives to explain contract cheating, investigating students’ motivations for why they cheat, or why they do not (Rundle et al., 2019), the prevalence and character of the problem, including likelihood of recidivism (Curtis & Clare, 2017), its wider socio-cultural and institutional determinants (see Smith et al., 2013; Nagy & Groves, 2021), and the capacity of institutions and their staff to detect and respond through crime prevention (Clare et al., 2017). Some have examined opportunity-based theories, identifying that while efforts to reduce opportunities for cheating are relevant to prevention, they form only part of effective intervention efforts and must be complemented by strategies targeting student psychology and motivation (Baird & Clare, 2017; Rundle et al., 2019). The authors (see Nagy & Groves, 2021) recently noted that engagement in contract cheating, and academic misconduct generally, is typically linked to an assemblage of events, pressures, settings and opportunities, representative of a form of *strained rationality* (a meshing and parallel application of rational choice and strain theories) that is often beyond students’ control. A feature of that study was its acknowledgment of the role that criminology and/or criminal justice can play in reducing cheating through evaluation of its various individual, socio-cultural, structural, and institutional determinants, for which it is well-situated to offer prevention strategies to address the *causes* of offending (Nagy & Groves, 2021).

### Legislative Attempts to Curb Contract Cheating

Several jurisdictions have enacted legislation in some form to respond to the problem of contract cheating, including New Zealand, Ireland, 17 states in the US (see Amigud & Dawson, 2020) and now Australia, the latter discussed shortly. At the time of writing (May 2021), MPs in the UK are debating whether and how existing laws might be amended to restrict contract cheating, specifically its provision and advertisement (Turner, 2021; QAA, 2017). New Zealand sought to address contract cheating in 2011 through amendments to its *Education Act 1989* (s292E), which criminalised the provision or advertisement of cheating services, also establishing a penalty framework of fines, up to NZD10,000, applied to service providers but not students (Draper & Newton, 2017). Ireland has also made recent legislative changes, established through the *Qualifications and Quality Assurance (Education and Training) (Amendment) Act 2019 s43a,* which afford its regulatory body, Quality and Qualifications Ireland (QQI), statutory powers to prosecute anyone engaging in cheating activities (QQI, 2019). No prosecutions have been made using this law since its establishment (Irish Independent, 2020). As Draper and Newton (2017:3) summarise “some countries/states have laws designed to prevent the activities of contract cheating services, but they have not been very effective”.

### Legislative reform: the Australian experience

In September 2020, the *TEQSAA(PACS) Act 2020* (Cth) was passed, making it an offence to provide or advertise academic cheating services in HE. The Act offers a series of amendments, including articulation of a further primary objective: “to protect and enhance the academic integrity of courses provided by higher education providers by prohibiting academic cheating services” (s3ss(g)). The legislation also expands TEQSA’s responsibilities, to include a key prevention role regarding the provision and advertisement of cheating services (*TEQSA Act 2011*, part 1, division 2, s4). Specifically, TEQSA can now seek court injunctions to force Internet service providers (ISPs) and search engines (e.g., Google) to block contract cheating websites or those that advertise such services. This power extends to the collection and dissemination of information about cheating websites and their users to help institutions respond to cheating on campus.

**Fig. 1:**
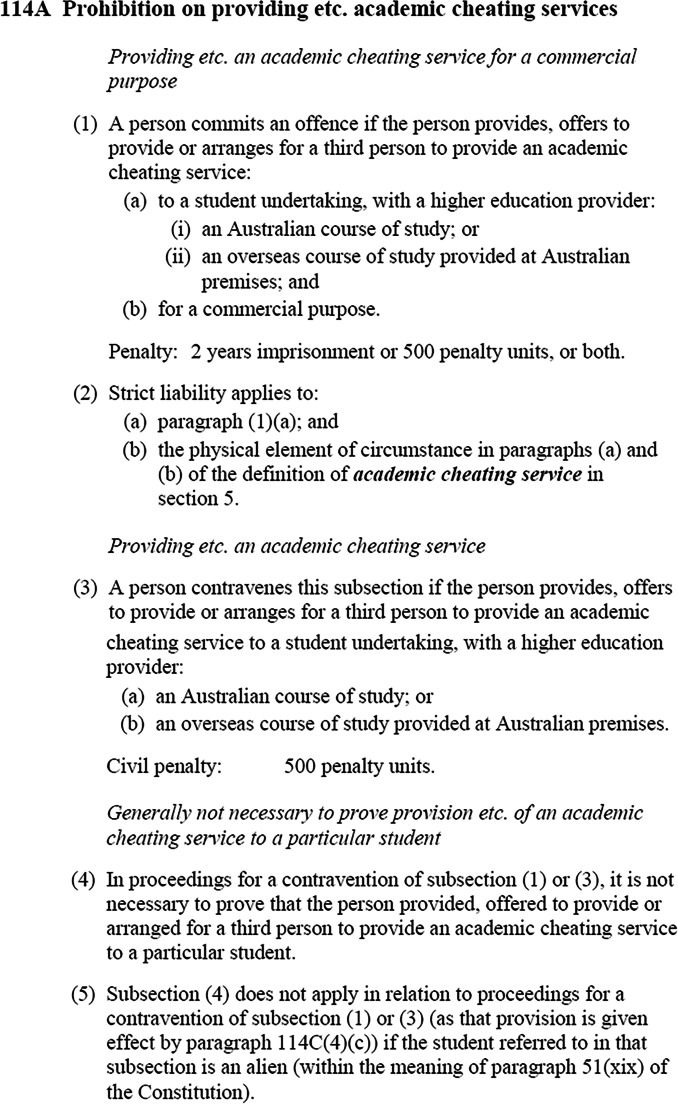
Amendments to *TEQSA Act 2011*, related to provision of cheating services: s114A

**Fig. 2:**
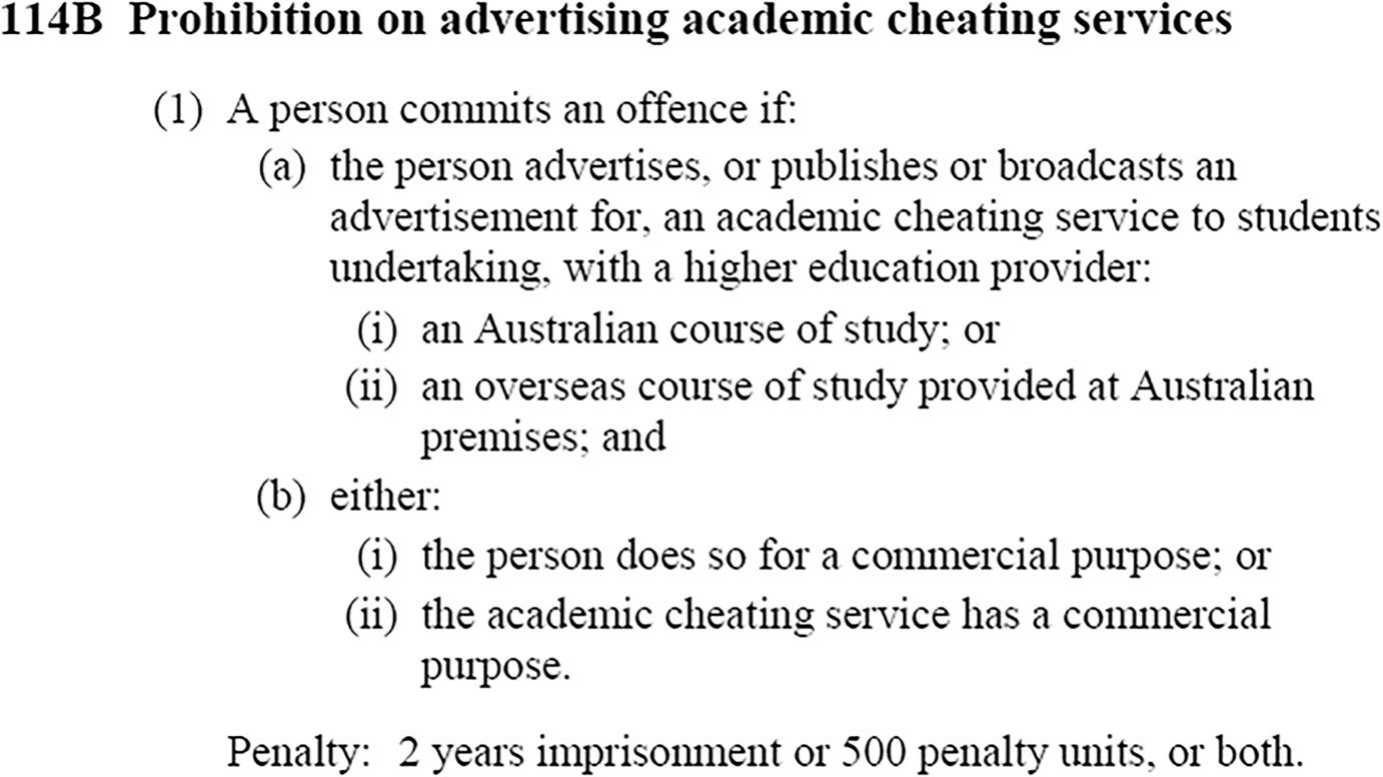
Amendments to *TEQSA Act 2011*, related to advertisement of cheating services: s114B

The legislation separates provision of cheating services (see Fig. 1) from their advertisement (see Fig. 2), as they each relate to students undertaking studies with an Australian university or an overseas course of study provided at Australian premises. This serves to recognise the “continually changing contract cheating marketplace”, including those who provide cheating services and the ‘enabling sites’ that advertise them (Lancaster, 2020:12).

**Fig. 3:**
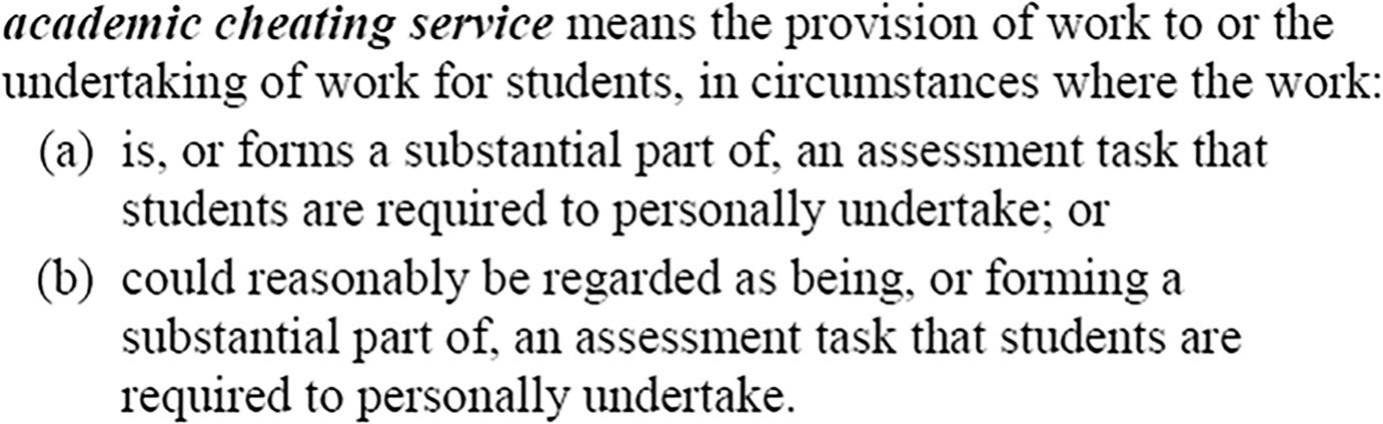
Amendments to *TEQSA Act 2011*, definition of academic cheating services: s3, ss5

The *TEQSAA(PACS) Act 2020* also defines ‘academic cheating service’ (see Fig. 3), perhaps one of the first examples of legislation to do so, which aligns with those in the academic literature (Clarke & Lancaster, 2006; Clare et al., 2017; Awdry, 2020), as well as many institutions’ academic misconduct policies (see Deakin University, 2019). This provision addresses one of the concerns outlined in debates in New Zealand, Ireland, and the UK (see Draper & Newton, 2017; Irish Legal News, 2017). The second, arguably more significant, element of the Australian reforms is its inclusion of a strict liability clause (see s114A, ss(2)) in relation to the provision of cheating services. Essentially, an offence of strict liability is committed when the evidence obtained supports the commission of said offence, without the requirement to prove intent/intention on the part of the offender. Intent has been a particularly contentious aspect of the international debate regarding cheating legislation, raising challenges as to the effectiveness of the New Zealand reforms and delaying the UK laws (see, for discussion, Draper & Newton, 2017:5; Draper et al., 2017).

A further feature of the *TEQSAA(PACS) Act 2020* amendments, is that they target cheating services, provided, or advertised for either commercial or non-commercial purposes. Alongside the fact that the offences and penalties the Act establishes will apply whether services are provided and/or advertised domestically or from overseas, this may not only be ineffective but also have potentially significant unintended consequences (Amigud & Dawson, 2020). This clause is particularly notable (s114A, ss3), as it captures the myriad ways students outsource their work, which more often involves assistance from other students, friends, and family without any form of monetary payment or commercial arrangement (Awdry, 2020). Even though the possible net-widening of this legislation has been identified and defended within parliamentary debates, where Dan Tehan (LNP), then Minister for Education, stated that “the clear intention with this bill is to deter cheating assistance rather than to prosecute friends and family members” (Hansard, 4 December 2018, 6913), there is insufficient protection against it. Instead, it is possible that such ‘providers’ may face penalties of up to two years’ imprisonment and fines of up to AUD100,000 for criminal breaches (those with a commercial purpose) and a AUD100,000 fine for civil breaches (those considered non-commercial).

There are several ideological and/or philosophical concerns with current forms of cheating legislation that should trouble criminologists and communities alike. For instance, exposure of a wider population (e.g., students, their friends and parents) to the criminal justice system and its related harms represents a form of ‘net widening’ (see Austin & Krisberg, 1981; Braithwaite, 1989) prominent in debates within New Zealand, Australia and the UK, where – in line with philosophies of prevention and diversion – it is emphasised that students should not be the targets of criminalisation (Nagy & Groves, 2021). However, the primary objection raised by this research is that the legislation is not problem-oriented, and does not seek to address the causes of, or motivations for students’ engagement in contract cheating. That there remain hundreds of websites offering cheating services indicates a demand for what they offer, the determinants of which are unlikely to be reduced by criminalisation of third-party services alone (Nagy & Groves, 2021; Draper & Newton, 2017). More importantly, analysis of the political discourse reveals many and varied ‘problems’ indicative of a much more complex and intractable debate regarding contract cheating. We argue this is a characteristic of crime discourses in general, where contemporary problems (or fears) are used strategically by governments and politicians, allowing them to discuss whatever they like with impunity and representing opportunities to be seen to be ‘doing something’ about crime and justice issues regardless of the efficacy of proposed ‘solutions’. In this analysis of Australian political discourse, the construction of contract cheating as a crime through the *TEQSAA(PACS) Act 2020* enables the othering and exclusion of international students through racialised narratives, the marketing of ‘Australianness' as a mechanism of reinforcing national interests generally while also justifying the state’s intervention in HE, as well as paying lip service to the ‘vulnerabilities’ of university students in Australian HE. The criminalisation of contract cheating in the Australian context then, is characteristic of paternalistic states where problems are not fully known or agreed upon, which means that very few are likely to be solved by reforms such as this legislation.

## Approach and materials

### Political Discourse Analysis and Interpretive Policy Analysis

In this paper, we combine Political Discourse Analysis (PDA) and Interpretive Policy Analysis (IPA) to explore parliamentary debates around the criminalisation of contract cheating services. This decision was made as the immediate and wider contexts (including the socio-cultural and historical) of the political debates (associated with contract cheating here) needed to be considered (Filardo-Llamas & Boyd, 2017), not just what was said by Federal Members of Parliament (MPs) and Senators. Attention on political debate as an avenue for discourse analysis for criminologists is beneficial because texts created by politicians ‘beckons further study due to the very nature of politics itself’ (Filardo-Llamas & Boyd, 2017:323); that is, it is public, impacts a large proportion of the population, and are the words and actions of those elected to represent the wider citizenry. In the case of legislation that is criminal, and carries punitive sanctions, criminology has a role to play in examining the purposes of such legislation as well as, in due course, how it has been policed and enacted.

Similarly, IPA is interested in the wider contexts within which the text is located. However, attention is on the performative aspect of policy. As Yanow (2000) has argued, when policymakers communicate or debate policy and the resulting recommendations, then the text contains their symbolic values and beliefs. Policy debate is therefore performative: it is both part of the policy process (e.g., parliamentary debate) and the producers of the policy (e.g., party ideology). IPA examines not just what is meant but how it is meant, that is, the processes through which policy meanings are conveyed and how this is interpreted by the intended audiences. Wash (2020) demonstrates that discourse analysis is increasingly accepted when discussing education policy because it can allow for research and analysis into ways that personal, party political and institutional ideas and beliefs can shape public policy. This aligns broadly with what Eagley and Chaiken (1993) previously highlighted, that social actors act and communicate as members of social movements or ideological groups with shared attitudes about social issues. This creates a strong case then, for not only policy but also political, and especially parliamentary debate being analysed using a framework that is underpinned by a discursive framework.

### Australian Parliamentary Hansards

We examined parliamentary debates in the Australian Federal Parliament which took place in 2019/2020. These debates are transcribed and presented in Hansard, a verbatim record of the official debates had within the two houses of Parliament (the House of Representatives or the Lower House, and the Senate or the Upper House). The debates were linked to the *TEQSAA(PACS) Bill 2019*. In Australia, the current Federal Government is a Liberal/National Party (LNP) Coalition which is traditionally situated as a centre-right, while the Opposition is the Australian Labor Party (ALP), which is considered a centre-left party. Also, in opposition in both houses of Parliament are several minor parties such as the Greens (left), the One Nation Party (right), Centre Alliance (centrist) and independents.

We have focused exclusively on analysing the Australian debates about introducing the legislation linked to prohibiting academic contract cheating services. There are several reasons for this. Firstly, the Australian debate was more in-depth and protracted than debates in Ireland and New Zealand, where for the latter Hansard records were hard to locate, with debate seemingly less overt and mostly part of much broader reforms to the HE industry during 2017/2018. By comparison, the Irish debate was incredibly limited, and the proposed legislation was largely related to efforts to “empower QQI as a regulator of quality and strengthen the Agency’s roles in ensuring high standard across Ireland’s education system” (Seanad Eireann debate, 10 Oct 2018). Therefore, the current paper examines the Australian debate through a more targeted lens, despite the issue of contract cheating being transnational. With efforts underway to introduce similar legislation in the US and the UK, it is valuable and timely for criminology to be involved in analysis of government moves to widen the net and enact new legislation that arguably treads the same ground as existing laws.

## Findings

### The ‘Dragon in the Room’: Chinese University Students

In introducing the Bill, then Minister of Education, Dan Tehan (LNP) stated that the need for this legislation was because.

international education is one of Australia’s biggest export industries and this bill demonstrates to potential students, their parents, their governments and also to overseas employers that Australia takes the quality and integrity of its higher education system and graduates very seriously (Hansard, 4 December 2019, 6913).

Unsurprising to anyone currently working in Australian HE is the Federal Government’s underfunding of Australian universities coupled together with university Vice-Chancellors driving to increase international student numbers and retention. International students are responsible for at least 26% of Australian Universities’ revenue (Horne, 2020). Concurrently to the COVID-19 pandemic, the locking of JobKeeper (unemployment benefits for those who lost jobs due to the pandemic) to prevent sessional or part-time university staff from accessing it, and the closing of borders to international arrivals, the relations between the Chinese and Australian governments have also continued to deteriorate (Wadhwa, 2021). With the largest portion of international students arriving from China, the issue of how Australia engages with one its largest trading powers and the effects on Australian HE has come to a head, played out within the debates about contract cheating.

Parliamentary discussions about the Bill quickly descended into debate about international students, honing-in on two major points: Australia’s over-reliance on international students and the other, that China specifically was exerting too much influence with examples of Chinese government interference on Australian university campuses (Hurst, 2021). The mentions of international students could be read as a euphemism for Chinese students, as in 2018 there were 205,189 Chinese students enrolled in Australian universities, followed by students from India comprising a further 89,570 out of a total of over 690,000 international students to Australia (DET 2018). However, Australia is a popular destination for students also from Nepal, Brazil, Malaysia, Colombia, Sri Lanka, Philippines, Bangladesh, Chile, Kenya, and Mongolia (DET 2018). Where international students are mentioned in the debates it cannot be said that these refer exclusively to Chinese students, however, we argue that based on the tone of the discussions and explicit mention of the Chinese Communist Party’s (CCP) influence in Australian HE, this indicates shared attitudes amongst parliamentarians about China’s strength and influence on this facet of Australia’s economy.

With regards to Australian HE and over-reliance on foreign nationals, three key themes emerged: the over-reliance on these students for funding purposes, the vulnerability of these students in Australia, and finally the economic cost to Australia of contract cheating. Themes around economic cost were associated with LNP politicians, while concern with vulnerable students was associated with ALP politicians. Other minor party parliamentarians did not contribute responses relevant to either theme.

Unsurprisingly, the issue of protecting Australia’s economic interests saturated statements made by LNP politicians, who took foreign investment in HE to be a positive feature, with statements revealing that the Bill was an ‘important step in protecting the value of our international students and the value of our higher education’ (Hansard, 12 June 2020, 4099, Allen [LNP]), or that HE ‘is one of Australia’s biggest export industries’ (Hansard, 12 June 2020, 3004, Birmingham [LNP]). Others from the LNP were critical of universities for appealing to and for international students, and instead praised the Bill for ‘bring[ing] to light many different aspects of our tertiary sector, not just its over-reliance on foreign students [at a time when] we fund the sector a record high levels’ (Hansard, 24 August 2020, 104, Molan [LNP]). The tackling of contract cheating through this legislation was seen as one way to protect any ‘significant negative impact on Australia’s economy’ and ‘future education exports’ (Hansard, 24 August 2020, 110, Rennick [LNP]) if left uncontrolled. As an export commodity therefore, it was also labelled vital to ‘maintain confidence’ by international markets (Hansard, 12 June 2020, 4099, Allen [LNP]). Senator Henderson (LNP) linked lax contract cheating legislation to the possible detriment of Australian business and ‘the prosperity of our country’ (Hansard, 24 August 2020, 116). Likewise, Senator Rennick (LNP) was concerned that previous instances of contract cheating, especially the MyMaster scandal, ‘revealed that the integrity and quality of Australia’s higher education was brought into question in the minds of foreign regulators’ (Hansard, 24 August 2020, 110).

Conversely ALP politicians used this debate as an opportunity to discuss how universities were eager for international students as funding avenues, due to LNP policies. Senator Carr did not mince words when stating.

international student fees fund research at Australian universities, but the people who complain about the reliance on the fees have so far done nothing to provide a more secure basis of funding by the Australian Government, where the funding should actually come from (Hansard, 24 August 2020, 113).

However, Carr was alone in being this explicit in their presentation of the issues of university funding in Australia. Instead, ALP speakers predominantly focused their attention on the vulnerability of students, especially international students, who may be more likely to employ contract cheating services. The economic cost of studying and living in Australia for many of these students was highlighted by MP Ryan (ALP) who argued that together with the exploitation of international students in employment ‘many students are unable to give their studies appropriate attention’ (Hansard, 12 June 2020, 4105). Senator Bilyk (ALP) concentrated on social vulnerabilities, ‘particularly …for international students who may be away from family and friends for the first time’ (Hansard, 26 August 2020, 3). The link between vulnerability and university practices was highlighted by MP Perrett (ALP), who was concerned that the English-language skills of many students who resorted to contract cheating were low but ultimately responsibility lay with the universities for enrolling them (Hansard 12 June 2020, 4096). Senator Ayres encapsulated the ALP position best when stating that there was a ‘solemn contract’ between the Australian government, HE, and parents which meant money for the education of the students in exchange for looking after them; failure to uphold this contract had led to Australia’s actions during COVID-19 being labelled by Ayres a ‘disgrace’ (Hansard, 24 August 2020, 106).

Explicitly framing this legislation as a question of Chinese influence on Australian campuses was part of statements made about international students and their student fees de facto funding HE. LNP politicians such as Henderson, Molan, Chandler and Rennick framed their concerns as being ‘about free academic inquiry on campus’ at a time when the ‘influence of the CCP on university campuses’ was on the rise (Hansard, 24 August 2020, 114, Chandler [LNP]). Molan and Rennick both referenced the Drew Pavlou case at the University of Queensland to illustrate the insidious nature of the CCP (Hansard, 24 August 2020, 105–109). Pavlou was suspended from his studies in 2019 after a protest at the University of Queensland turned violent. Pavlou was protesting the human rights record of the CCP and the treatment of the Uighur population in China and had received death threats from an adjunct professor who was also a Chinese Consul worker. A senate inquiry is currently underway into university courses being financially paid for by the CCP, the recruitment of Australian academics for Chinese research programs, and interference and intimidation of staff and students on Australian university campuses. Senator Henderson went so far as to argue that the Bill, in conjunction with the Foreign Influence Transparency Scheme legislation to prevent espionage and foreign interference in Australia, would safeguard Australia, intellectual property, and national security (Hansard, 24 August 2020, 116, Henderson [LNP]). The reliance on Chinese students especially but foreign students in general has led to universities losing ‘popular support in Australia’ (Hansard, 24 August 2020, 104, Molan [LNP]), in effect placing the blame for the need for contract cheating legislation at the feet of universities.

The only non-LNP parliamentarian to discuss Australian-Chinese relations was Senator Carr (ALP). The debate quickly moved from support for the Bill to lambasting government for being ‘quite happy to acquiesce to having our university scholars pilloried by its own backbenches, because it is easier to get a headline by inciting fear about security breaches’ (Hansard, 24 August 2020, 113, Carr [ALP]). While Carr was defending the HE sector, it quickly became apparent that for both MPs and Senators the contract cheating legislation was an opportunity to air political grievances rather than focus on the issue of academic misconduct.

### Political Point-scoring

This debate is replete with examples of political point-scoring, which speak to the broader politicisation of Australian HE. It is not surprising, nor new, that debates regarding law reform are a likely setting of point-scoring from all sides of politics, especially when considering social issues of crime and misconduct. However, the ways in which these points have been ‘scored’ provide unique insight into the political context of Australia’s tertiary sector. Furthermore, these parochial, emotive, and often divisive narratives drill down into the core of the politics of crime and criminal justice.

While there was broad support for the need to ‘crack down’ on contract cheating, evident in the bipartisan support for the Bill, the Hansard also revealed several other concerns that shape HE and that overshadowed or pushed-aside discussion of contract cheating. MP Ryan (ALP), for example, was “… appalled that the *real issues* confronting vocational and higher education in this country … are left fallow for another term of this government” (Hansard, 12 June 2020, 4107, emphasis added). These concerns can be categorised into three groups, as they relate to funding cuts, threats to academic values and freedoms, and the practical constraints on universities and their staff that limit their capacity to function effectively, which collectively constrain opportunities for Australia’s intellectual, economic, and social prosperity.

#### Funding cuts and COVID

A prominent feature was discussion of the nature and scope of funding/resource cuts to HE and its implications for the many stakeholders who rely on this industry. Most members in this debate mentioned funding, either lauding their own party’s efforts to increase support or denigrating their opponents’ desire for ‘cuts’. For example, members of the Coalition Government extolled “that the federal government gives $17 billion in funding to higher education” (Hansard, 24 August 2020, 109, Rennick [LNP]). MP Murphy (ALP) countered, claiming that:

By capping university places, cutting $2.2 billion from the system and locking more than 200,000 students out of the opportunity of a university qualification, this government is doing the current generation and future generations of Australians a massive disservice. Cutting $328.5 million from university research is something to be ashamed of (Hansard, 12 June 2020, 4100).

The impacts of these cuts were not considered specific to HE, but indicative of a much wider concern about reduced government support – particularly for international students (Perrett (ALP) Hansard, 12 June 2020, 4096).

This attack was not limited to the experiences of or consequences for international students with many politicians also worried about the apparent funding cuts and reduced access to services and universities, for domestic students and their families, including those from rural and regional communities. A sense of abandonment of rural Australians was conveyed by Labor, with MP Ryan (ALP) railing that “the impact on regional communities will be devastating. Universities support 14,000 jobs in country Australia—that's tens of thousands of livelihoods destroyed” (Hansard, 12 June 2020, 4106). Senator Ayres (ALP) likewise criticised the LNP Government, reporting that “there is a confused position in the National Party, which should be standing up for regional universities” (Hansard, 24 August 2020, 107).

The impacts of funding cuts were depicted as widely felt, concurrently linked to employment and economic prosperity, as well as core principles of nationhood and social identity, given Australia’s reputation as the land of opportunity (Sydney Morning Herald, 2005). However, the current situation was presented very differently by the respective political parties. On one hand, Australia’s educational future was viewed positively, with the sector considered “literally a plethora of opportunity” (Hansard, 12 June 2020, 4099, Allen [LNP]). On the other, Senator Bilyk (ALP) was dismayed that:

Universities are economic powerhouses within the community, particularly in our regions. They provide jobs, train regional workers and prepare our young people for the future challenges that our country is facing and will face. … Yet, when it comes to our higher education system, this government's approach is cut, cut, cut (Hansard, 26 August 2020, 3).

Perhaps the most significant example was the intense politicking associated with COVID-19, the timing of which coincided with much of the period of debate for the Bill (March-September 2020). In addition to emphasising the importance of jobs, university funding and the role of HE in ensuring ongoing prosperity, there was an intense focus on JobKeeper, the Coalition Government’s financial support package created to meet the challenges posed by COVID and subsequent response strategies (i.e., lockdowns, etc.).

Some claimed JobKeeper represented the Government’s “…absolute lack of commitment to the sector overall” (Hansard, 12 June 2020, 4105, Ryan [ALP]), while others depicted a more divisive, intentional strategy to point-score:

…the Morrison government has gone out of its way to exclude universities from some of the COVID support programs. The Morrison government has repeatedly changed its policy … to stop university staff from accessing wage subsidies, and it is putting thousands of jobs at risk (Hansard, 12 June 2020, 4096, Perrett [ALP]).

I urge this government, as it is looking at JobKeeper, to look at two groups who have been left out that are relevant to this legislation—that is, vulnerable students and casuals who haven't been in the same workplace for 12 months. Many of the people I've spoken about are young students who are working to support themselves … to get through university and, of course, the universities themselves (Hansard, 12 June 2020, 4100, Murphy [ALP]).

It is not the purpose of this paper to examine the legitimacy or accuracy of the figures cited, promises made or effectiveness of policies implemented. However, collectively, these issues highlight the increased risk of contract cheating, with significant pressures placed on the HE sector, its institutions, staff and – undoubtedly – its students.

#### Threats to academic freedoms/values

Debate also homed in on the need for legislation to account for threats to academic freedom and values, with topics ranging from the principles of academic integrity to policy inclusivity to freedom of speech. Debate was driven by a more direct recognition of the consequences for academic integrity, with Senator Faruqi noting:

… academic integrity is best protected, in the first instance, by adequately funding education and training providers, where low student-staff ratios allow staff to ensure integrity by developing an individual understanding of each student's abilities in order to detect and address issues before they become misconduct. … Yet this government is set upon cutting further funding from universities and hiking fees for students. It's absolutely abhorrent and shameful. It is cruel and callous (Hansard, 24 August 2020, 104 [Greens]).

The focus on Government decisions to cap numbers, increase HECS debts (federal government loans to university students) and reduce repayment thresholds, as well as increase fees for certain courses (e.g., Arts and Humanities) was seen by many as a political opportunity to exclude certain, already vulnerable groups of the community. It was noted that students from low-income backgrounds, first-in-family students, and international students – those most at risk of engaging in contract cheating (Bretag et al., 2019) – are limited in their academic choices and opportunities by these policy decisions:

People struggle at university. We know this. We also know that people from vulnerable and disadvantaged backgrounds particularly struggle at university. Many people go to university as the first person in their family to do so, and it has been a matter of aspiration and hard work and sacrifice to get there. … So it's easy to see how vulnerable students who don't have a support network around them can be targeted by these professional organisations for cheating (Hansard, 12 June 2020, 4100, Murphy [ALP]).

Further demonstrating partisan politics, discussion of the Bill was also used to convey concerns about academic freedoms more broadly defined and the role of universities, particularly by members of Government. Senator Molan (LNP) noted universities’.

… overreliance on foreign students, remembering that the minister reminded the sector only recently that their primary function is to educate Australian students and that anything else is up to them and is their responsibility. … the consequence of so many foreign students is that our universities have lost their Australianness … Australian national interests must come first, and universities, it seems to me, are losing popular support in Australia (Hansard, 24 August 2020, 104).

Senator Molan further questioned the sector and its regulatory body, TEQSA, using the debate to highlight LNP concerns about political ideology and the failure of universities to protect academic freedoms:

When I think of Australian universities today, I don't think of them as places of learning where intellectual freedom thrives. If that were the concept that drove our universities, a student guild running stalls for new students wouldn't dream of banning a right-wing Generation Liberty stall on the basis that its brand did not align with the guild's values, regardless of the spin. If intellectual freedom were taken seriously, a vice-chancellor would not put up with this rubbish on their campus; neither would the regulator nor our government (Hansard, 24 August 2020, 104).

These sentiments were countered by the opposition, with Senator Ayres (ALP) shortly thereafter reflecting on the attempted point-scoring by noting “I can say that nothing illustrates more fully how lost the coalition is on these issues than the obscurantism of Senator Molan's most recent comments on this issue” (Hansard, 24 August 2020, 106).

#### Neo-liberalisation and tertiary capacity

Flowing from concerns regarding funding cuts and lack of adequate resourcing, further doubt was cast about the nature of contemporary HE and its capacity (or support) to produce critical ideas and high-quality research, while ensuring academic integrity. Within the subtext of COVID-19, Opposition members were concerned about the neo-liberalisation of HE and the consequences of a business model of tertiary pedagogy, with some noting that “This anti-intellectual government has done everything in its power to silence and diminish the influence of our experts” (Hansard, 12 June 2020, 4106, Ryan [ALP]). Ryan described the culture that this approach fosters, remarking:

… the fact that we need a bill like this to deter students from accessing cheating services saddens me as an educator. We've turned our places of higher learning into factories for jobs. Kids are there to get the piece of paper that will help them end up in any office that will give them a consistent pay cheque. That's not the kind of academic culture that will foster the innovation this country needs … (Hansard, 12 June 2020, 4106).

These comments fit with a broader theme of structural change within HE, which has emerged from Government concerns that “…it is becoming increasingly clear that education in Australia has been on the decline for the past 20 years” (Hansard, 24 August 2020, 109, Rennick [LNP]). This has laid the groundwork for support of a business model to HE, driven by principles of neo-liberalism. Senator Hughes (LNP), highlights a discourse of competition, ‘job-readiness’ and success:

Australian and international university students are under more pressure than ever before to succeed. The pandemic and the fast-evolving digital business world mean that there is more competition than ever when it comes to applying for jobs. Qualifications could mean the difference between winning a role or not (Hansard, 25 August 2020, 17).

The consequences of current policies, particularly for students’ likelihood of engagement in contract cheating were clear:

We know that pressures like the financial consequences of failing a subject or academic requirements for visa retention are often present in cheating circumstances. This bill and the rest of the government's higher education policy settings do nothing to fix this. … All of these conditions have worsened during the pandemic, as the move to online learning has isolated students from opportunities to seek support and has limited academics' ability to assist a student before they begin to engage in academic misconduct” (Hansard, 24 August 2020, 104, Faruqi [Greens]).

These comments highlight not only the extent of and desire for political point-scoring, but also the consequences of such politicking, which counterintuitively limit the capacity of Australian tertiary institutions, their staff, and students to ward off the threat of contract cheating.

### A need to punish!

When debate did resume on the content of the Bill rather than on political talking points, all members agreed the legislation would protect students, deter wrongdoing, and ensure the reputation of Australian HE is upheld. Somewhat ironically, for a bill about academic cheating and misconduct, multiple speeches from LNP members were aimed at allaying the fears that the legislation would seek to punish parents, friends, and family for helping university students. Senator Antic stated this legislation was not aimed at ‘mums and dads at home who help proofread or edit a son’s or daughter’s essay or give advice on how to improve an assignment’ (Hansard, 24 August 2020, 110–111, Antic [LNP]). This was echoed almost verbatim by Senators Henderson, Molan, Birmingham, Hughes, and Rennick, demonstrating two key points. One, while the ministers and senators were aware of contract cheating, they were not aware that they were in fact endorsing academic collusion when speaking positively of parents editing their children’s work, and two, the lack of paraphrasing or even key differences in the speeches was amusing to the authors as it represents plagiarism.

A strongly held view among LNP members was that “it is important that we send a clear signal that cheating in higher education is not just immoral but also illegal” (Hansard, 12 June 2020, 3004, Birmingham [LNP]). The emphasis on immorality, and the fact cheating goes against our national identity and culture was palpable, with cheating described as “more than anything else … a corrosive behaviour which undermines the entirety of a society” (Hansard, 25 August 2020, 18, Scarr [LNP]). This moralising is particularly important, as it was used to justify not only criminalisation, but also the reach of the legislation, with other LNP members railing that “To not stamp out cheating services poses a significant threat to the integrity and the reputation of our higher education system, both in Australia and internationally” (Hansard, 24 August 2020, 111, Antic [LNP]). Reputational risk was a concern that seemingly unified MPs and Senators, alongside the associated risk of harm to the community. Senator Antic noted that contract cheating “irresponsibly put the lives of others at risk as a result of assumed knowledge and the possession of skills obtained during these courses” (Hansard, 24 August 2020, 111, Antic [LNP]). This was echoed by Senator Rennick (LNP) who was also concerned with public wellbeing from “graduates [who] are not thoroughly qualified for their jobs” (Hansard, 24 August 2020, 110, Rennick [LNP]). MP Hammond (LNP) also saw contract cheating as a risk to employers (Hansard, 12 June 2020, 4104). Such risk thinking is significant, as it justifies a more punitive approach to crime and offending that has permeated much of criminal justice debate, politicking and policymaking in Australia in the last few decades (Cherney & Sutton, 2007). However, ‘tough on crime’ rhetoric is considered largely incompatible with the principles of crime prevention (Cherney & Sutton, 2007), highlighting a paradox in this arguably political debate that justifies investigation of the motivations for such punitive discourse and policymaking regarding contract cheating.

Despite the acknowledgement that many forms of cheating are already subject to criminal and civil penalties tied to other offences, such as fraud or misrepresentation, it was argued that “these can be complex and difficult to pursue. … They also provide little deterrence, as there is no specific law that clearly and simply says the provision of cheating assistance is wrong” (Hansard, 12 June 2020, 3003, Birmingham [LNP]). The need for re-tooling or more heavily ‘arming’ TEQSA with specific cheating legislation was extolled by many LNP members, as well as several across the floor (e.g., Ryan, Perrett, Pratt, Bilyk [ALP]), to address what one considered “…possibly the greatest current reputational risk to Australian higher education” (Hansard, 26 August 2020, 2, Bilyk [ALP]). Given the common perception that the extant laws did not work or were too onerous, it is unsurprising that calls for new/additional legislation to catch and punish cheaters were loud.

In addition to supporting the creation of new legal mechanisms, these calls also provided TEQSA with the capacity to enforce more severe penalties on offenders, for the purpose of *deterring* those engaged in contract cheating. Deterrence was a consistent theme, with many articulating “…the maximum penalties in this bill have deliberately been set high to create a strong deterrent to the provision and promotion of cheating services” (Hansard, 12 June 2020, 3004, Birmingham [LNP]). This was recognised by the Education Minister, who rationalised that “while these penalties are severe, they are consistent with those for comparable offences such as dealing in fraudulent identity information…” (Hansard, 4 December 2019, 6913, Tehan [LNP]). Labor “strongly support[ed] the deterrence measures” (Hansard, 24 August 2020, 102, Pratt [ALP]), at least for commercial service providers, but was very concerned about the consequences for students and families, wanting to:

make sure that the legislation doesn't implicate vulnerable students who have received no personal benefit from their actions or are ignorant of what these services actually involve (Hansard, 12 June 2020, 4096, Perrett [ALP]).

A key observation regarding these measures and the attitudes to them, consistent across political lines, was the assumption that greater penalties will have commensurate deterrent effect. Senator Griff from the Centre Alliance (CA) lauded “The severity of these penalties is designed to act as a deterrent and I'm confident that they will have the desired effect” (Hansard, 24 August 2020, 108). Similarly, Senator Birmingham (LNP) claimed that the legislation and penalties aim to “significantly change the incentives for commercial operations” (Hansard, 12 June 2020, 3004). However, the deterrent effect of this, and similar, legislation on contract cheating is difficult to determine (Draper & Newton, 2017). Indeed, it should be noted that to date (December 2021), the legislation has only been employed once, in May 2021, with TEQSA seeking to use the legislation to force 51 Internet service providers to block Australian students from accessing an alleged cheating website ‘Assignmenthelp4you.com’ (Taylor, 2021). Notably, this case only utilises one facet of the legislation focusing on attempts to block access, while there is currently no indication that the regulator is seeking to apply the criminal penalties (imprisonment or heavy fines) also enshrined in the legislation and that were the feature of parliamentary debates.

## Discussion

Analysis of recent Australian parliamentary debate regarding the need for specific legislation to address the provision and/or advertisement of contract cheating services reveals a multifaceted, dynamic, and often divisive political inquiry tasked with identifying a solution to the cheating ‘problem’. The *TEQSAA(PACS) Act 2020* has a dual purpose, which seeks to reduce or prevent the provision *and* advertisement of cheating services by both commercial *and* non-commercial providers through threat of serious criminal penalties (imprisonment and fines). Our analysis of the discourses from debate of the initial Bill reveals an acknowledgement of the growing challenge posed by contract cheating and the widespread concern about the reputational risk to the integrity and quality of Australia’s HE, which the leaders of our nation argue must be stopped and offenders – *cheaters* – punished. While there is universal agreement that contract cheating represents a serious problem that needs to be fixed, there was – and continues to be – considerable disagreement on how this should be achieved.

In this paper, we identify two key findings that arguably shaped the nature and extent of debate, and the ensuing legislation. Firstly, ingrained in members’ comments, from all sides of politics, was the need to detect and prevent academic fraud because it is wrong. The desire to send the clear signal that cheating at university is not just morally wrong, but also legally, was built upon the criminological principle of deterrence and the assumption that threat of severe punishment will deter criminality (Akers, 1990). This ‘tough on crime’ attitude fits with the law-and-order approach typical of Australian politics across the last four decades, popular because it allows governments to “avoid appearing ‘soft’ on crime while gesturing in the direction of prevention” (Cherney & Sutton, 2007:71). However, a long history of research does not support this assumption, both in the study of crime generally (see Akers, 1990), as well as in empirical studies that investigated the influence of penalties on cheating behaviour (see Nagin & Pogarsky, 2003; Amigud & Dawson, 2020). As Amigud and Dawson (2020:106) have found, the “ability to deliver detection and enforcement is limited” whereby “supply-side approaches to the contract cheating problem are [not] likely to have any significant positive effects”.

Secondly, and more importantly, our analysis has identified widespread concerns that the legislation will have the effect of exposing university students, their peers, friends, and families to negative *and criminal* consequences. A major finding was that there exists a strong, parochial desire to punish those who cheat, even if this is at the cost of vulnerable, unaware, and unsupported students. All speakers, regardless of party affiliation, were supportive of legislation to protect vulnerable students from ‘predatory operations’ (Hansard, 12 June 2020, 4096, Perrett [ALP]). Reading through Hansard, however, an image of university students in Australia is unveiled: they are naïve, often constructed as international students ‘from Asia with poor English struggling with their courses’ (Hansard, 12 June 2020, 4099, Allen [LNP]) who may share details of contract cheating services without understanding that cheating is wrong, are away from home and under pressure with their studies, and who do not have parents or friends who could edit their work for them or understand what legitimate academic services look like. It was therefore unclear which students needed protection and why; those whose parents were editing their university work for them, or those who were from non-English speaking backgrounds and did not have parents who could edit their work for them. What is clear though, is that the language in Hansard was highly paternalistic, painting university students (especially foreign students) in Australian HE as incapable of understanding right from wrong, and often receiving help on their assignments from family and friends in order to pass. This is supported by the research evidence (Awdry, 2020), where it is reported that students more often seek assistance from social networks than commercial providers, which means that the legislation – at best – addresses only part of the problem.

The hegemonic discourse on this issue, as represented in the parliamentary debates, provided diverse perspectives on possible crime prevention methods to address contract cheating. However, like universities, these largely focused on situational factors, such as reducing immediate or proximate opportunities for offending and not the ingrained, more deeply embedded problems. The legislation, and legislators, are looking for the ‘quick-fix’ option – not problem-oriented policymaking (Cherney, 2006, 2009). Much of the debate about the need for and importance of the Bill conveniently ignored who created the problem that the proposed legislation would fix. In the debate, politicians acknowledged that it is not the students, but that there are many external pressures that drive some students to engage in or feel the need to engage in alternative strategies to achieve academic success, which exposes them to exploitation by third-party providers. Essentially, this legislative approach is trying to ‘fix’ the problem without acknowledging what the problem is. A one-size-fits-all approach that does not contextualise the problem and does not consider the likely effectiveness of solutions is arguably short-sighted.

The criminalisation of contract cheating then, must be understood within a wider context of the politicisation of Australian HE, as a discursive construction that has significantly influenced when, how and why the legislation was introduced and debated. The Bill emerged at a time when a series of disparate efforts to regulate and restrict HE, its institutions, staff, and students seemingly coalesced, conveniently when Australians were simultaneously struck by the challenges of COVID-19. This allowed the debate to encompass both legitimate concerns about academic integrity and reputation, as well as thinly veiled xenophobia elicited by perceived threats to national security, nationalism, community safety, academic freedom, and social and economic independence. We therefore argue that the introduction of this legislation represents an opportunistic step by a myopic government, blinded by local political issues, where the criminalisation of contract cheating has engendered debate characteristic of general crime discourses and that obfuscates real problem(s). Moreover, this framing has allowed discussion of several, arguably spurious political talking points that have served to other, exclude, and neglect the needs of vulnerable students, reinforce national interests and notions of ‘Australianness’, and rationalise paternalistic state intervention through greater regulation of students in Australian HE, despite the acceptance of cheating as a transnational problem. As articulated by Senator Faruqi (Greens) (Hansard, 24 August 2020, 103), this is a “punitive response to academic misconduct”, which compounds the extant challenges of increased monitoring and punishment of students using surveillance software (Zhou, 2020), the removal of HECS support from students who fail more than half their subjects (MacMillan, 2020) and selective fee increases for certain courses of study not considered ‘relevant’ (Duffy, 2020).

Considerable recent research, including critical Australian studies, emphasise the need to address the myriad strains, pressures and expectations placed on university students that establish the demand for cheating services (Clare et al., 2017; Amigud & Dawson, 2020; Awdry, 2020; Nagy & Groves, 2021). We suggest that the recent Australian legislation is misdirected and misses an important opportunity to address contract cheating and academic misconduct generally, by examining both third-party service provision and how it is related to student performance and tertiary pedagogy, greater understanding of which may provide further avenues for intervention and prevention. There is need to focus on contract cheating at the ‘coal face’, to implement proactive, targeted, and educative situational prevention initiatives relevant to students and their learning environment, rather than waiting until they reach out to or are lured by predatory service providers and punishing all parties if/when cheating is detected and successfully prosecuted (however likely).

## Conclusion

Contract cheating is a problem that, if left unregulated, threatens the integrity and reputation of Australia's HE industry, both domestically and internationally. However, tough on crime rhetoric only extends so far in its capacity to foster meaningful change, which means that for efforts to reduce and prevent cheating to work, detailed exploration of the underlying motivations for and broader influences and contexts of contract cheating is needed. This is already underway, but more is needed. While we recognise that the criminal law may have value in supporting the detection and sanction of large-scale commercial cheating service providers, it is only one of many strategies that must be recognised and implemented if contract cheating is to be reduced. Despite the claims of its supporters, we question if the *TEQSAA(PACS) Act 2020* (Cth) represents a genuine attempt to address contract cheating, though only time will determine whether it has value or if it is just an ‘Act’.

## Data Availability

Not applicable.
